# Mpox outbreak in Abia State, Nigeria, 2024: implications for varicella zoster virus coinfection among children

**DOI:** 10.11604/pamj.supp.2025.50.1.47524

**Published:** 2025-12-05

**Authors:** Chidinma Ihuoma Amuzie, Ugochukwu Uchenna Onyeonoro, Princess Orie Agomoh, Elezuo Okorie Elezuo, Stanley Ajamgbulogu, Oluchi Adighogu, Princewill Chigozirim Isaac, Chukwuma David Umeokonkwo

**Affiliations:** 1 Department of Community Medicine, Federal Medical Centre, Umuahia, Abia State, Nigeria,; 2 Department of Public Health and Disease Control, Abia State Ministry of Health, Abia State, Nigeria,; 3 Nigeria Centre for Disease Control and Prevention, Abuja, Nigeria,; 4 African Field Epidemiology Network, Kampala, Uganda

**Keywords:** Case, epidemiology, Mpox, Nigeria, outbreak, poxviridae

## Abstract

**Introduction:**

Mpox remains a significant public health concern in Africa with a notable increase in cases and outbreaks in several countries. Despite the efforts of surveillance and other specific interventions, Nigeria has been reporting ongoing cases since its re-emergence in 2017. We conducted an epidemiologic description of the 2024 Mpox outbreak in Abia State to understand the drivers and inform its control strategies.

**Methods:**

we investigated the cases and conducted a descriptive study of suspected cases of Mpox line listed in 2024. Data were collected with a standardized case investigation form. An active case search was conducted in affected communities, and a review of hospital records was done. Samples were collected and tested at the National Reference Laboratory for confirmation. The data was downloaded from SORMAS, cleaned, and analysed using SPSS. Frequencies, proportions, and attack rates were generated.

**Results:**

sixty-one suspected Mpox cases were line listed and 10 (16.4%) were laboratory-confirmed, with 6(60%) out of the confirmed cases having varicella zoster virus (VZV) coinfection. Among the confirmed cases, males constituted 60% (6/10), and the median age was 9.5 years (IQR: 3 - 27 years). The predominant age group was 0 - 15 years, 7 (70%). Out of the 12 reporting LGAs, confirmed cases were dominant in 5 LGAs. The commonest symptoms were fever (90%) and itchy vesiculopustular rash (90%). The most common symptom was fever (30%). Among all confirmed cases with rashes (90%), skin lesions were mostly on the face, legs, arms, and genitals. Ukwa East LGA had the highest attack rate (3/100,000 population).

**Conclusion:**

the confirmed cases were mostly children with a high prevalence of VZV coinfection, and males were mostly affected. We recommended targeted public health awareness campaigns to the demographic groups mostly affected and emphasized enhanced surveillance and early diagnosis to minimize complications from Mpox/VZV coinfection.

## Introduction

Mpox is a reemerging viral infectious disease endemic in Central and West Africa [1] and poses a significant global public health threat. The virus belongs to the family of Poxviridae, the subfamily of Chordopoxvirinae based on its animal host, and to the Orthopoxvirus genus, including variola virus, cowpox virus, and vaccinia virus [2]. It is known to have two clades - Clade I (subclade Ia and Ib) and Clade II (subclade IIa and IIb) [3]. Clade I is more virulent and is responsible for human-to-human transmission. It is a zoonotic infection known to infect both animals and humans. In 1959, a pox-like disease outbreak was first reported in monkey colonies kept for research in Copenhagen, Denmark. The first human case was in a nine-month-old infant in the Republic of Congo [4]. Between 1971 and 1978, ten human cases were reported, and three confirmed in Nigeria, with the first index case in Abia State reported in 1971 [4]. Subsequently, there has been a history of Mpox occurrence in different African countries.

Since January 1st, 2022, cases of Mpox have been reported to the World Health Organization (WHO) from 25 member states across Africa, with most cases from the Democratic Republic of Congo, Burundi, and Uganda [5]. As of January 12th, 2025, 17,498 laboratory-confirmed cases, including 87 deaths, have been reported to WHO [5]. While Nigeria had reported 1,043 laboratory-confirmed cases, including 9 deaths, mainly due to the Clade II MPXV strain [5]. Interventions from WHO and subnational responses to curtail the outbreak include coordinating research on vaccines and treatments, strengthening country health systems, improving access to vaccines, therapeutics, and diagnostics, and developing the SORMAS (Surveillance Outbreak Response Management and Analysis System) to enhance in-country surveillance, among others [3,6]. In 2022-2023, a global outbreak of Mpox was caused by the clade IIb strain [3]. Consequently, in May 2022, the WHO declared the MPX outbreak a Public Health Emergency of International Concern, and again in August 2024, for the second time due to the burden of the disease [3].

Nigeria experienced the largest outbreak of Mpox in 2017, caused by the West African clade, affecting several states in the country, including Abia State [6]. With the increase in the magnitude of the disease, human Mpox cases were exported from Nigeria to Israel and the United Kingdom (UK) in 2018, and to Singapore in 2019 [7]. Furthermore, these cases marked the first human cases exported from Africa and were linked to the first confirmed human-to-human transmission outside Africa through a nosocomial infection in the UK [7]. Studies from different zones of Nigeria have documented varying patterns and clinical presentation of the disease, suggesting varying human dynamics and notable demographic shifts. Studies have demonstrated that Mpox tends to occur more often among males and adults [4,8]. Another study has also reported that it primarily affects children [9]. It has been reported to be predominantly more common among some specific demographic groups, including men who have sex with men (MSM), bisexual individuals, and HIV-positive individuals [10-12]. Furthermore, it has been observed that rash is the most predominant symptom, and most times precedes fever [10,11]. Additionally, most skin lesions have been documented to appear more often on the face [6,10,13]. Although Mpox outbreaks have occurred in Abia State, there remains a significant paucity of detailed epidemiological data describing the disease within the region. We conducted an epidemiologic description of the 2024 Mpox outbreak in Abia State to understand the drivers and inform its control strategies

## Methods

**Study design and area:** this is a cross-sectional study of the suspected and confirmed cases of Mpox reported from January to December 2024. Abia State is one of the southeastern states in Nigeria, with 17 Local Government Areas (LGAs). Most urban LGAs in the state contain slums. Based on a 2.7% annual growth rate, the state’s population was projected to reach 4,577,915 in 2024, up from the 2006 national census. Most of the land areas are densely populated with thick forests, and the primary occupation of the residents is agriculture, including wildlife activities and trade. There is a history of Mpox cases, which are usually reported in the neighboring states of Enugu, Akwa Ibom, Imo, and Rivers. There are designated isolation and treatment centres for Mpox in the state. However, there are no laboratories in the state equipped to conduct Mpox testing. The Mpox outbreak response is being coordinated by the Epidemiology Unit of the Public Health Department, Abia State Ministry of Health, in collaboration with key partners. These include the World Health Organization (WHO), which supports surveillance activities, and the Nigerian Red Cross Society, which is focused on public awareness and community sensitization. At the LGA level, the response activities are supervised by the LG Disease Surveillance Notification Officers (DSNO).

**Study population:** this included all reported Mpox cases in the State, encompassing suspected, probable, and confirmed cases. A case was classified based on the National public health response guidelines [14]. They included:

**Suspected case:** an acute illness with fever > 38.3°C, intense headache, lymphadenopathy, back pain, myalgia, and intense asthenia followed one to three days later by a progressively developing rash often beginning on the face (most dense) then spreading elsewhere on the body, including soles of feet and palms of hand.

**Probable case:** a case that meets the clinical case definition, is not laboratory confirmed, but has an epidemiological link to a confirmed case.

**Confirmed case:** a clinically compatible case that is clinically confirmed.

**Sample size determination:** the sample for this study consisted of all 61 reported cases of Mpox from January to December 2024 in Abia State. Cases of Mpox not reported to the LGA or State Surveillance unit were excluded from the study

**Study tool and data collection:** suspected Mpox cases reported in all Local Government Areas (LGAs) during the study period were investigated by the State Ministry of Health’s Epidemiology Unit, in collaboration with Local Government teams. Standardized case investigation forms provided by the NCDC were used for this process [14]. The form captured data on socio-demographics, medical history, clinical signs and symptoms, exposure history, and contact information on humans and animals. Vesicular swabs, crust, and whole blood were collected from each eligible case by the LG laboratory focal person. Before collecting the samples, the swab containers and blood tubes were labeled with the case’s name, date, age, sex, and EPID number. Hand hygiene and the recommended personal protective equipment (PPE) were appropriately used following established guidelines before collecting the sample [14]. The samples collected were then forwarded to the state laboratory officer. At the state level, the samples were prepared for transportation using the triple packaging according to the guidelines [14]. The samples were then transported to the National Reference Laboratory in FCT Abuja, along with copies of the laboratory forms, using third-party logistics provided by the Nigeria Centre for Disease Control (NCDC) for laboratory confirmation. Also, the cold chain temperature was maintained throughout the process. Laboratory confirmation of Mpox and varicella-zoster virus infections was performed using the real-time polymerase chain reaction (RT-PCR) assay on vesicular swabs and crust samples. The surveillance officer extracted and uploaded information from the case investigation forms into SORMAS. Results of the laboratory investigations were also uploaded to the SORMAS. In Nigeria, SORMAS has been adapted as the tool for eIDSR (electronic IDSR) and provides real-time information for immediate action [15].

### Operational definition

Mpox/VZV coinfection: this was defined as the detection of MPXV-specific DNA and VZV-specific DNA by polymerase chain reaction in the tested sample.

**Statistical analysis:** the finalized data was then extracted from SORMAS in an Excel format. The data was cleaned and coded, and imported into SPSS version 26 for analysis. Univariate analysis was used to describe the socio-demographic information, exposure characteristics, and the clinical presentation of symptoms of the confirmed cases. Continuous variables, such as age, were summarized using the median and Interquartile range (IQR), while categorical variables were presented as frequencies and percentages. Missing values were noted as unknown in the dataset. QGIS Desktop 2.18 was used to plot the person map.

**Ethical consideration:** ethical clearance was obtained from the Health Ethics Committee of the Abia State Ministry of Health (reference number: AB/MH/PRS/ECS/T.1/436), and the ethical principles outlined in the Declaration of Helsinki were adhered to during this research. Also, permission to access the data was obtained from the Department of Public Health, Abia State Ministry of Health. To ensure confidentiality, all identifiers were removed, and all collected data were stored securely with access limited to the principal investigator.

## Results

**Demographic and exposure characteristics of all confirmed Mpox cases:** ten cases were laboratory confirmed as Mpox (16.4%) out of a total of 61 suspected cases listed. Among the confirmed cases, the median age was 9.5 years (IQR: 3 - 27 years), and a greater proportion (70.0%) of the confirmed cases were within the age group of 0 - 15 years. The proportion of Mpox/VZV coinfection was 60% (6/10), out of which 3 (50%) were in the 0 - 4 years category, followed by 2 (33.3%) in the 10 - 14 age group, and only 1(16.7%) within the 5 - 9 age range. Among the Mpox/VZV coinfections, the median age was 5.5 years (IQR: 4.0 - 11.0). Males constituted 60% of all the confirmed cases. Most of the confirmed cases were students/pupils (70.0%), and only 40% of them were exposed to a case. There was no history of exposure to animals among all the confirmed cases Table 1.

**Table 1:** demographic and exposure characteristics of the confirmed Mpox cases, Abia State, Nigeria, 2024

Variable	Frequency	Proportion (%)
**Age (all confirmed cases)**		
0 - 15	7	70.0
>15	3	30.0
**Median (IQR)**	**9.5 (3.0 - 27.0)**	
**Mpox/VZV coinfection**		
Yes	6	60.0
No	4	40.0
**Age (mpox/VZV coinfection) n=6**		
0 - 4	3	30.0
5 - 9	1	10.0
10 -14	2	20.0
**Median (IQR)**	**5.5 (4.0 - 11.0)**	
**Sex**		
Male	6	60.0
Female	4	40.0
**Occupation**		
Student/pupil	7	70.0
Civil Servant	1	10.0
Trading	1	10.0
Tailoring	1	10.0
**History of contact with a case**		
Yes	4	40.0
No	6	60.0

IQR: interquartile range; VZV: varicella zoster virus

**Figure 1:**
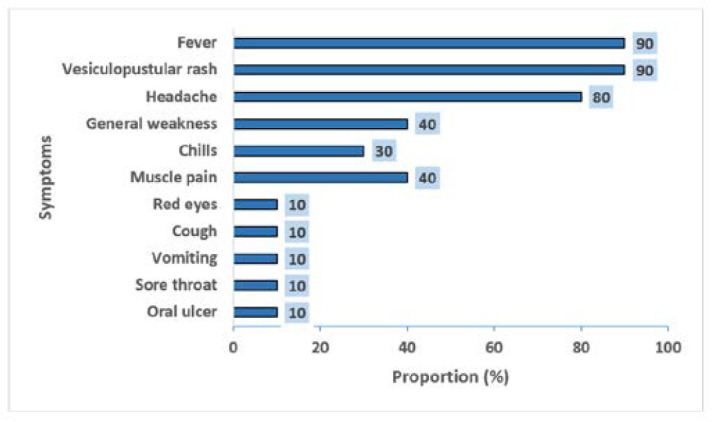
distribution of symptoms among the confirmed cases of Mpox in Abia State, Nigeria, 2024

**Distribution of symptoms among the Mpox confirmed cases:** fever was the most prevalent preceding symptom (30%) among the confirmed cases. Additionally, the commonest symptoms reported by the confirmed cases included fever (90%) and itchy vesiculopustular rash (90%), followed by headache (80%). The least prevalent symptoms included oral ulcer (10%), sore throat (10%), amongst others Figure 1.

**Characteristics of rashes among the Mpox confirmed cases:** almost all the confirmed cases (90%) had rashes on the faces, legs, arms, genitals, and all over the body. Three-fifths of the confirmed cases observed the rash on the soles of their feet. Among all the confirmed cases who had rash, 88.9% of them had rashes that were profound, while two-thirds of them had rash in the same stage of development and of the same size Table 2.

**Table 2:** characteristics of rashes among the mpox confirmed cases in Abia State, Nigeria, 2024

Variable	Frequency	Proportion (%)
**Location of rash**		
Face	9	90.0
Legs	9	90.0
Arms	9	90.0
Palms	8	80.0
Genitals	9	90.0
Thorax	8	80.0
Sole of the feet	6	60.0
All over the body	9	90.0
**Description of rash (n=9)**		
Rash the same size	6	66.7
Rash lesion profound	8	88.9
Same state of development	6	66.7

**Figure 2:**
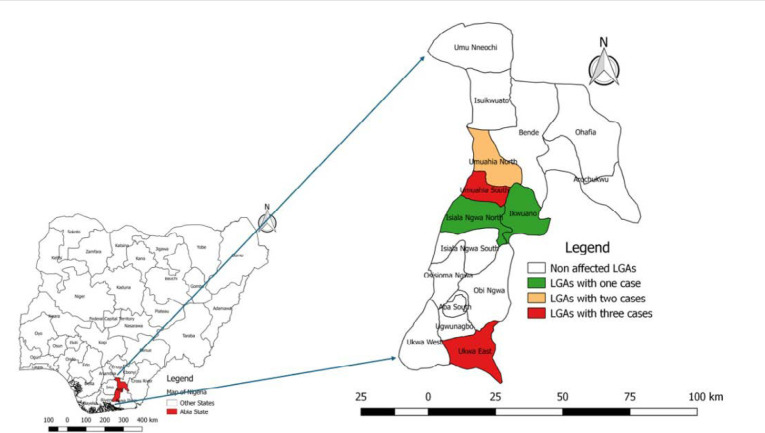
spatial distribution of confirmed cases of Mpox by LGAs in Abia State, Nigeria, 2024

**Spatial distribution of confirmed Mpox cases:** five out of 12 reporting LGAs had at least one laboratory-confirmed case of Mpox, with the highest number of confirmed cases (3) in Umahia South and Ukwa East. The highest attack rate in the population was recorded in Ukwa East, with 3 cases per 100,000 population, compared to other LGAs Figure 2.

## Discussion

We provided an epidemiologic description of the 2024 Mpox outbreak in Abia State to understand the drivers and inform its control strategies. The Mpox/VZV coinfection was high and prevalent among children. Confirmed cases of Mpox were more common among males and children. Most skin lesions were seen on the face and extremities. Also, the common symptoms at presentation were fever and vesiculopustular rash, with fever being the preceding symptom.

We observed a high level of VZV coinfection rate among the Mpox confirmed cases, and all the cases of coinfection were children less than 15 years old. This finding elucidates the endemicity of chickenpox in Nigeria, where VZV vaccination coverage is abysmally poor [12]. A study has postulated that chickenpox is a risk factor for Mpox in Nigeria [16] with other parts of West and Central Africa [17], implying that Mpox eradication will be difficult among children. The existence of both illnesses often leads to misdiagnosis, especially in resource-constrained settings where laboratory facilities are not readily available for immediate testing, as well as the increase in turnaround time. The delay in proper diagnosis can affect the appropriate public health measures to be instituted and may lead to a waste of resources. Also, it is noted that severe clinical presentations occur in Mpox/VZV coinfection, which can result in prolonged illness and more complications compared to those affected with only Mpox [11,18]. The VZV coinfection rate observed in our study was higher than the rates reported in Nigerian studies [9,11,18,19] and DRC [20,21]. This is concurrent with the finding of a study conducted in South-south Nigeria, where the odds of Mpox positivity were higher in children with VZV infection [12]. However, this contrasts with the result of a study in Nigeria, where adults predominated the chickenpox cases [18].

A study in DRC postulated that initial infection with either Mpox or Chickenpox virus predisposes humans to be susceptible to a secondary infection, alternatively, that Mpox infection directly triggers VZV infection [22]. This could be supported by the findings of a very low VZV seroprevalence in a previous serosurvey in DRC [23]. In Nigeria, Chickenpox is not a notifiable disease, and its vaccine is not part of the routine immunization vaccines in the National Programme on Immunization Schedule (NPI). Therefore, this calls for the institution of a robust surveillance system to track the trend of chickenpox infection and prevent cases of chickenpox. Also, the surveillance system should be supported by intense risk communication to ensure early presentation and avoid delayed diagnosis, as well as a high index of suspicion among healthcare providers.

Males were mostly affected in this study. This aligns with the findings of most studies conducted in Nigeria [6,12,18]. However, a recent study in Congo revealed that females, particularly those who were sex workers, were most affected [24]. This is likely more due to transmission dynamics than the inherent susceptibility of the disease. Moreover, recent trends in most outbreaks predominantly involved males who were gay or bisexual. A significant proportion of cases may occur within this group due to skin-to-skin contact during intimate activities. Also, males are more exposed to wildlife reservoirs through their hunting lifestyle, predisposing them to the animal host. Though our population, mostly children under 15 years old, were not found to be hunters, and most of them did not have previous contact with a known case. Furthermore, women tend to have better health-seeking behaviour compared to men, who are less likely to seek early medical attention, leading to increased spread. There is a need for targeted preventive strategies, particularly among the high-risk male populations.

In this study, children were the most affected, and this finding resonates with a study conducted in North-eastern Nigeria [9]. However, several studies describing the epidemiological trend of Mpox have documented that adults were mostly affected [4,6,8,10,25]. Children are part of the vulnerable population at higher risk of severe illness and complications [3]. Earlier epidemics in Africa showed that children less than 15 years old were the most affected [1,21]. This may be associated with the reduction in herd immunity following the discontinuation of the smallpox vaccination. It is noted that clade I MPXV transmission in DRC is more commonly reported among children [26]. In Nigeria, with more prevalent clade II cases, transmission among children would be enhanced by the presence of zoonotic reservoirs, overcrowding aiding close contact, and inadequate hand and environmental hygienic conditions. There is a need to prioritize childhood Mpox vaccination within the program.

Most cases presented with skin lesions primarily on the face and extremities, exhibiting the centripetal rash distribution pattern that has been previously documented in Mpox cases. This finding is consistent with earlier research, which highlights that rashes appeared all over the body, with more predominance on the face [6,13,27]. Also, in this study, fever was the prominent symptom and preceded rash. This was similar to a study done in Nigeria [13]. Conversely, in other earlier studies conducted in Nigeria, rash was the first presenting symptom [9-11]. This varying pattern predicts the epidemiological shifts in findings earlier reported. This necessitates continuous monitoring of the symptoms typically reported by suspected cases to refine the diagnostic expertise of the attending healthcare professional.

A few limitations were observed in this study; firstly, temporality could not be ascertained due to the type of study design. Secondly, we had a few variables and a small sample size for further analysis, as this was a secondary data source. This could affect the generalizability of our findings to the general populace. Finally, the study was limited to only one year, so we could not ascertain the trends of Mpox cases over the years in Abia State. Notwithstanding, the strength of this study lies in the fact that it gives an insight into the varying epidemiology of Mpox, generates hypotheses for future research, and informs interventions aimed at controlling Mpox in Abia State, Nigeria.

**Conclusion**

A total of ten laboratory-confirmed cases were reported, with a high proportion of Mpox/VZV coinfection. Confirmed cases were primarily males, and children were mostly affected. Additionally, all cases with Mpox/VZV coinfection were in the pediatric population. The most common symptom was fever, with skin lesions predominantly located on the face and extremities. We recommend awareness campaigns and community sensitization initiatives specifically designed for high-risk demographic groups within schools, workplaces, and other critical settings. Childhood Mpox vaccination should be prioritized among the population mapped out for Mpox vaccination in Nigeria. Also, the intensification of Mpox and chickenpox surveillance is vital for early detection and monitoring. Furthermore, it is essential to reorient healthcare workers on the current patterns of Mpox symptoms to refine their diagnostic proficiency.

### What this study adds

- Major outbreaks have been observed following the resurgence of human mpox in Nigeria, reported in 2017; this comes 40 years since Nigeria last reported a case;
- The disease primarily affects men, adults, and people with HIV infection.

### What is known about this topic

- Six out of ten confirmed cases had concomitant VZV infection;
- All the Mpox/VZV coinfections belonged to the pediatric population.

## References

[1] Jezek Z, Marennikova SS, Mutumbo M, Nakano JH, Paluku KM, Szczeniowski M (1986). Human monkeypox: a study of 2,510 contacts of 214 patients. Journal of infectious diseases.

[2] Alakunle E, Moens U, Nchinda G, Okeke MI (2020). Monkeypox Virus in Nigeria: Infection Biology, Epidemiology, and Evolution. Viruses.

[3] World Health Organization (2024). Mpox. WHO.

[4] Faye O, Pratt CB, Faye M, Fall G, Chitty JA, Diagne MM (2018). Genomic characterisation of human monkeypox virus in Nigeria. Lancet Infect Dis.

[5] WHO Global Mpox Trends. World Health Organisation (WHO).

[6] Yinka-Ogunleye A, Aruna O, Dalhat M, Ogoina D, McCollum A, Disu Y (2019). Outbreak of human monkeypox in Nigeria in 2017-18: a clinical and epidemiological report. Lancet Infect Dis.

[7] Mauldin MAM (2022). Exportation of monkeypox virus from the African continent. J Infect Dis.

[8] Chieloka OS, Bammani IM, Amao LK (2022). Descriptive epidemiology of the burden of human monkeypox in Nigeria: a retrospective review 2017-2021. Pan Afr Med J.

[9] Stephen R, Alele F, Olumoh J, Tyndall J, Okeke MI, Adegboye O (2022). The epidemiological trend of monkeypox and monkeypox-varicella zoster viruses co-infection in North-Eastern Nigeria. Front Public Health.

[10] Ogoina D, Iroezindu M, James HI, Oladokun R, Yinka-Ogunleye A, Wakama P (2020). Clinical Course and Outcome of Human Monkeypox in Nigeria. Clin Infect Dis.

[11] Ogoina D, Dalhat MM, Denue BA, Okowa M, Chika-Igwenyi NM, Yusuff HA (2023). Clinical characteristics and predictors of human mpox outcome during the 2022 outbreak in Nigeria: a cohort study. Lancet Infect Dis.

[12] Ogoina D, Dalhat MM, Denue BA, Okowa M, Chika-Igwenyi NM, Oiwoh SO (2024). Mpox Epidemiology and Risk Factors, Nigeria, 2022. Emerg Infect Dis.

[13] Akar S, Adesola YO, Akar S, Burga J, Oluwafemi B, Akinrogbe J (2020). Descriptive epidemiology of monkeypox in Nigeria, September 2017-June 2019. Int J Infect Dis.

[14] Nigerian Centre for Disease Control (2019). National Monkeypox Public health response guidelines. NCDC.

[15] Nigerian Centre for Disease Control National Technical Guidelines for Integrated Disease Surveillance and Response.

[16] Grose C (2023). Surveillance of Nigerian children suggests that varicella may be a risk factor for acquisition of monkeypox. Front Public Heal.

[17] Khallafallah O, Grose C (2022). Reassessment of Evidence about Coinfection of Chickenpox and Monkeypox (Mpox) in African Children. Viruses.

[18] Mmerem JI, Umenzekwe CC, Johnson SM, Onukak AE, Chika-Igwenyi NM, Chukwu SK (2024). Mpox and Chickenpox Coinfection: Case Series From Southern Nigeria. J Infect Dis.

[19] Onyeaghala C, Somiari A, Ichechiek U, Ogan J, Avundaa H, Ehiakhamen O (2025). Epidemiology, clinical presentation and outcome of human Mpox in Rivers State, Nigeria during the 2022-23 outbreak: an observational retrospective study. Pan Afr Med J.

[20] Hoff NA, Morier DS, Kisalu NK, Johnston SC, Doshi RH, Hensley LE (2017). Varicella Coinfection in Patients with Active Monkeypox in the Democratic Republic of the Congo. Ecohealth.

[21] Rimoin AW, Mulembakani PM, Johnston SC, Lloyd Smith JO, Kisalu NK, Kinkela TL (2010). Major increase in human monkeypox incidence 30 years after smallpox vaccination campaigns cease in the Democratic Republic of Congo. Proc Natl Acad Sci.

[22] Hughes CM, Liu L, Davidson WB, Radford KW, Wilkins K, Monroe B (2021). A Tale of Two Viruses: Coinfections of Monkeypox and Varicella Zoster Virus in the Democratic Republic of Congo. Am J Trop Med Hyg.

[23] Doshi RH, Alfonso VH, Mukadi P, Hoff NA, Gerber S, Bwaka A (2018). Low Varicella Zoster Virus Seroprevalence Among Young Children in the Democratic Republic of the Congo. Pediatr Infect Dis J.

[24] Vakaniaki EH, Kacita C, Kinganda-Lusamaki E, O´Toole Á, Wawina-Bokalanga T, Mukadi-Bamuleka D (2024). Sustained human outbreak of a new MPXV clade I lineage in eastern Democratic Republic of the Congo. Nat Med.

[25] Yinka-Ogunleye A, Aruna O, Ogoina D, Aworabhi N, Eteng W, Badaru S (2018). Reemergence of Human Monkeypox in Nigeria, 2017. Emerg Infect Dis.

[26] McQuiston JH, Luce R, Kazadi DM, Bwangandu CN, Mbala-Kingebeni P, Anderson M (2024). US Preparedness and Response to Increasing Clade I Mpox Cases in the Democratic Republic of the Congo-United States, 2024. Morb Mortal Wkly Rep.

[27] Ogoina D, Izibewule JH, Ogunleye A, Ederiane E, Anebonam U, Neni A (2019). The 2017 human monkeypox outbreak in Nigeria—Report of outbreak experience and response in the Niger Delta University Teaching Hospital, Bayelsa State, Nigeria. PLoS One.

